# A model for simulating the active dispersal of juvenile sea turtles with a case study on western Pacific leatherback turtles

**DOI:** 10.1371/journal.pone.0181595

**Published:** 2017-07-26

**Authors:** Philippe Gaspar, Maxime Lalire

**Affiliations:** Sustainable Management of Marine Resources, Collecte Localisation Satellite, Ramonville-Saint-Agne, France; Deakin University, AUSTRALIA

## Abstract

Oceanic currents are known to broadly shape the dispersal of juvenile sea turtles during their pelagic stage. Accordingly, simple passive drift models are widely used to investigate the distribution at sea of various juvenile sea turtle populations. However, evidence is growing that juveniles do not drift purely passively but also display some swimming activity likely directed towards favorable habitats. We therefore present here a novel Sea Turtle Active Movement Model (STAMM) in which juvenile sea turtles actively disperse under the combined effects of oceanic currents and habitat-driven movements. This model applies to all sea turtle species but is calibrated here for leatherback turtles (*Dermochelys coriacea*). It is first tested in a simulation of the active dispersal of juveniles originating from Jamursba-Medi, a main nesting beach of the western Pacific leatherback population. Dispersal into the North Pacific Ocean is specifically investigated. Simulation results demonstrate that, while oceanic currents broadly shape the dispersal area, modeled habitat-driven movements strongly structure the spatial and temporal distribution of juveniles within this area. In particular, these movements lead juveniles to gather in the North Pacific Transition Zone (NPTZ) and to undertake seasonal north-south migrations. More surprisingly, juveniles in the NPTZ are simulated to swim mostly towards west which considerably slows down their progression towards the American west coast. This increases their residence time, and hence the risk of interactions with fisheries, in the central and eastern part of the North Pacific basin. Simulated habitat-driven movements also strongly reduce the risk of cold-induced mortality. This risk appears to be larger among the juveniles that rapidly circulate into the Kuroshio than among those that first drift into the North Equatorial Counter Current (NECC). This mechanism might induce marked interannual variability in juvenile survival as the strength and position of the NECC are directly linked to El Niño activity.

## Introduction

Satellite tracking has uncovered the dispersal patterns of various adult sea turtle populations but, unfortunately, this is not the case for hatchlings and juveniles [1]. Juveniles are indeed much more rarely captured and tracked than adults while the tagging of small-sized hatchlings remains technically challenging and has been rarely achieved so far [2–4] The oceanic juvenile life stage of most sea turtle populations thus remains largely cryptic which seriously impedes the development of conservation measures focused on this critical life stage [5].

In that context, numerical models have become popular tools to simulate the movements and analyze the resulting spatial distribution of hatchlings and then juveniles. Most models assume that juveniles drift passively with ocean currents [6]. They are thus simple Individual Based Models (IBM) in which trajectories of thousands of particles, each representing a single individual, are simulated using readily available Lagrangian particle-tracking software fed with surface currents produced by ocean circulation models. These trajectories are then used to characterize the spatial distribution of the studied population and its evolution with time, e.g. [7–11].

However, evidence is growing that young sea turtles do not drift purely passively [4,12,13] and a few more elaborate IBMs have been developed to investigate the impact of active movements on juveniles’ dispersal patterns. Quite surprisingly, all models of this type focus on the impact of occasional movements such as oriented movements elicited by specific values of the Earth magnetic field [14,15], or movements occurring during the brief frenzy or post-frenzy period [16]. But none of these models deals with the, likely more usual, habitat-driven movements triggered by the need to find food and suitable water temperatures.

The goal of this paper is thus to develop a simple IBM simulating the dispersal of juvenile sea turtles under the combined effects of oceanic currents and habitat-driven movements. After presenting a generic version of this model, we parameterize it specifically for leatherback turtles (*Dermochelys coriacea*) and test its impact on the simulated dispersal of juveniles from the Western Pacific leatherback population. The passive dispersal of this population has already been investigated by Gaspar et al. [10] (hereafter GAL) which gives us a solid comparison basis to assess the various consequences of simulated habitat-driven movements on the dispersal and, ultimately, the life history of these juveniles.

## Materials and methods

### Model description

Our Sea Turtle Active Movement Model (STAMM) borrows ideas from previously developed fish movement and habitat models. In particular, the movement itself is modeled following Faugeras and Maury [17] while the habitat parameterization is similar to that of the SEAPODYM model [18]. While SEAPODYM was originally developed to simulate the spatial and temporal evolution of the density distribution of various age cohorts of a tuna population, a simplified, non age-structured, version of this model was successfully implemented to simulate the active dispersal of a single cohort of juvenile loggerhead turtles (*Caretta caretta*) in the North Pacific [19]. We will not follow this Eulerian modeling approach but will rather stick to the more flexible IBM (or Lagrangian) approach which has been largely used for passive drift modeling. A distinct advantage of IBMs is that they allow continuous updating of the age of each individual without the need to define distinct cohorts. More generally, IBMs, unlike Eulerian models, are designed take into account the effects of individual properties (such as sex, natal area, fitness,…) and naturally enable behavioral plasticity inside the same cohort. This shall be an advantage when further developing STAMM to simulate adults’ behavior and movements, including reproductive migrations.

#### Movement model

Habitat-driven movements are, by definition, triggered by the need of individuals to find, and stay in, most suitable habitats. They are therefore expected to possess the following characteristics:

1. In the absence of any clear habitat gradient, the movement shall be close to a random walk (no preferred direction);
2. As habitat gradients increase, the movement shall become more directed and lead individuals towards more favorable areas;
3. The movement speed shall decrease with habitat suitability so that individuals move rapidly through poor habitats and slow down in favorable zones.

**Fig 1:**
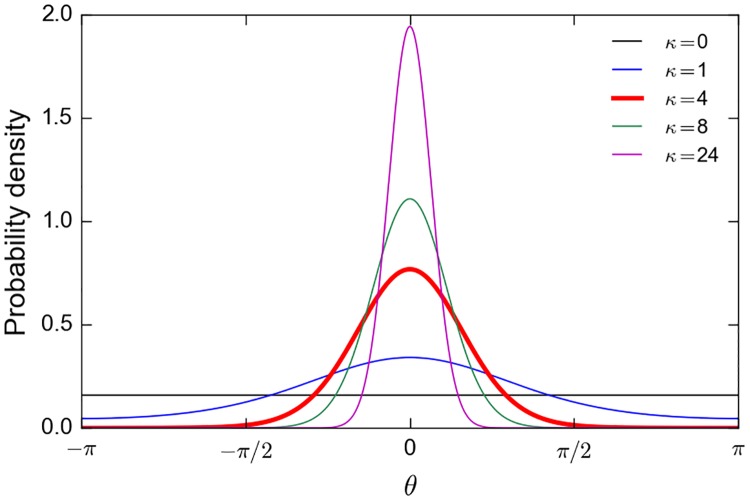
von Mises probability density function. The density is plotted for μ = 0 and different values of the concentration parameter κ.

Movements with characteristics (i) to (iii) are, quite easily and efficiently, simulated using the biased random walk model proposed by Faugeras and Maury [17]:

$$V_{s}\left(x,y,t,a\right)=V_{m}\left(a\right)\left(1-h\right)\;d$$ (1)

where ***V***_***s***_(*x*,*y*,*t*,*a*) is the habitat-driven horizontal swimming velocity vector of an individual of age a at position (*x*, *y)* and time *t*, *V*_*m*_ is its maximum sustainable speed, *h* is a normalized habitat suitability index (0≤ *h* ≤1) and ***d*** is the unit vector pointing in the direction of movement:

d=(cosθ,sinθ) (2)

where *θ* is the heading angle (relative to North). This angle is taken to be a realization of a stochastic variable having a von Mises distribution *νM*(μ, κ) with mean direction angle μ and concentration parameter κ. This distribution (Fig 1) is a circular analogue to the normal distribution. It converges to the uniform distribution as κ → 0 and tends to the point distribution concentrated in the mean direction μ as κ → ∞ (e.g. [20]).

The (1 − *h*) factor in Eq (1) guarantees that the swimming speed *V*_*s*_ (i.e. the norm of the velocity vector ***V***_***s***_) reaches its maximum value *V*_*m*_ in least suitable habitats (*h* = 0) and tends to zero in very favorable habitats (*h* →1) where individuals generally increase the time spent diving, searching for food, capturing and handling preys. Diving behavior however is not explicitly simulated.

The mean direction of movement is chosen to be the direction of the habitat gradient vector **∇*h***:

$$\mu =\theta_{\nabla h}$$ (3)

Modeled movements thus follow, on average, that gradient and hence tend to maximize habitat suitability. In addition, the concentration parameter κ is taken to be proportional to the norm of **∇*h***:

κ=α∥∇h∥ (4)

where α is a scaling parameter. Accordingly, when ∥**∇*h***∥ → 0, κ → 0 and the distribution of *θ* becomes uniform. On the contrary, large habitat gradients, and hence large κ values, yield strongly directed movement as the distribution of *θ* becomes strongly concentrated around the optimal direction *θ*_**∇h**_.

Interestingly, the simple movement Eq (1) proves to be the Lagrangian equivalent of an Eulerian advection-diffusion equation in which advection and diffusion are strongly linked and governed by habitat values and their gradients [17], as in the SEAPODYM model [18].

#### Estimation of the maximum sustainable speed

As implied by Eq (1), *V*_*m*_ is the speed at which a, likely fasting, animal will leave a very unfavorable area. It makes sense to assume that individuals escaping such areas will try to do so in the most energetically-efficient way, that is at a speed for which the amount of energy required to move one unit of distance, or work per meter (WPM), is minimum [21]. For an individual moving at speed *V*_*s*_, the work per meter is directly related to the rate of energy expenditure per unit time, that is the metabolic rate (*MR*):

$$WPM=MR/V_{s}$$ (5)

In a fasting individual, *MR* is the sum of the resting metabolic rate (*RMR*) plus the energy expended per second to move against the hydrodynamic drag force [22]:

$$MR=RMR+\rho \;C_{D}SV_{s}^{3}/\left(2\eta \right)$$ (6)

with *ρ* the sea water density, *C*_*D*_ the drag coefficient, *S* the surface of the turtle’s body and *η* is the overall efficiency coefficient of the flippers which includes their propeller efficiency and the aerobic efficiency of their muscles. Using Eq (6), Eq (5) can be rewritten:

$$WPM=RMR/V_{s}+\rho \;C_{D}SV_{s}^{2}/\left(2\eta \right)$$ (7)

Differentiating Eq (7) with respect to *V*_*s*_, the velocity that minimizes *WPM* is easily determined:

$$V_{m}={\left(\eta \;RMR/\rho \;C_{D}S\right)}^{1/3}$$ (8)

This velocity can then be expressed as a function of size (*L*) using simple allometric relations. Noting *M* the mass of the individual and assuming that *RMR* scales with *M*^*b*^ while *M* scales with *L*^*c*^ and *S* scales with *L*^*2*^, one obtains:

$$\;V_{m}=v_{0}\;L^{\frac{bc-2}{3}}$$ (9)

where *v*_0_ is a scaling parameter that remains to be determined. This equation governs the evolution of *V*_*m*_ with size, or with age provided that a growth curve *L*(*a*)is known for the modeled species.

#### Habitat model

Focusing on juvenile sea turtles looking for food and constrained by water temperatures, we express the habitat suitability index *h* as the product of a feeding habitat index (*h*_*F*_) and a thermal habitat index (*h*_*T*_) [18,19]:

$$h=h_{F}\;h_{T}$$ (10)

The feeding habitat suitability index, at a given place and time, is simply taken to be proportional to *P(x*,*y*,*t)* the local prey density (or a proxy of it), divided by the individual rate of food consumption *F* which varies with age (and species):

$$h_{F}\;\left(x,y,t,a\right)=Min\;\left[1,\;P\left(x,y,t\right)/\;F\left(a\right)\right]$$ (11)

Modulation of *h*_*F*_ by the rate of food consumption allows areas with relatively low abundance of preys to be favorable enough to support the foraging activity of young/small individuals while adults will seek richer areas.

**Fig 2:**
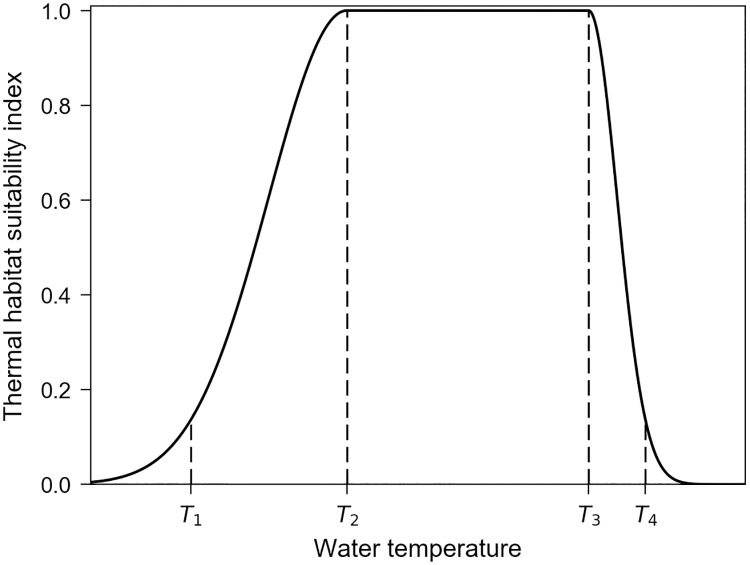
Thermal habitat suitability index as a function of water temperature.

Like all ectotherms, sea turtles can only perform in a limited range of body temperatures (*T*_*b*_). Because of the high thermal conductivity of water, *T*_*b*_ is closely linked to the surrounding water temperature (*T*_*w*_) so that sea turtles are forced to occupy a restricted range of water temperatures to avoid cold stunning or overheating. To model such a bounded thermal habitat we define 4 pivotal water temperatures: *T*_*1*_<*T*_*2*_<*T*_*3*_<*T*_*4*_ (Fig 2) where *T*_*1*_ and *T*_*4*_ are the critical temperatures below or above which an individual cannot survive for long while *T*_*2*_ and *T*_*3*_ are the lower and upper bounds of the thermal preferendum, that is the minimum and maximum water temperatures between which a sea turtle performs optimally or nearly so. The thermal habitat suitability index is then parameterized as:

$$\;\begin{array}{lll} h_{T}\left(x,y,t,a\right) & ={\text{ e}}^{-2\;{\left(\frac{T_{w}-T_{2}}{T_{2}-T_{1}}\right)}^{2}} & \text{if }T_{w}<T_{2}\\ & =1 & \text{if }T_{2}\leq T_{w}\leq T_{3}\\ & ={\text{ e}}^{-2\;{\left(\frac{T_{w}-T_{3}}{T_{4}-T_{3}}\right)}^{2}} & \text{if }T_{w}>T_{3} \end{array}$$ (12)

Of course, the pivotal water temperatures are species-dependent and can vary with mass, size or age. Note also that Eq (12) is formulated in such a way that when *T*_*w*_ reaches the critical values *T*_*1*_ or *T*_*4*_, *h*_*T*_ is close to zero but its gradient with respect to *T*_*w*_ remains significant. This ensures that individuals exposed to critical or near-critical temperatures will display vigorous movements directed towards more favorable thermal habitats.

### Model calibration for leatherback turtles

STAMM is fully defined by the equations determining the swimming velocity vector and the habitat suitability index. Its formulation is generic and can be used for any sea turtle species. To calibrate it specifically for leatherback turtles, we have to select the applicable allometric relationships and then calibrate the different parameters of the movement and habitat models.

#### Allometric relationships and movement model calibration

For leatherbacks, we assume that *RMR* scales with *M*^*0*.*831*^ [23] and use the growth curve and mass-length relationship of Jones et al. [24]:

$$L\left(a\right)\;=1.43\;\left[1-{\text{e}}^{-0.226\left(a+0.17\right)}\;\right]$$ (13)

$$M=112.31\;L^{2.86}$$ (14)

where *L* is the straight carapace length (SCL) in meters and *M* is in kilograms. These relationships imply *b* = 0.831 and *c* = 2.86, so that the relation defining the maximum sustainable speed Eq (9) reduces to:

$$V_{m}=v_{0}\;L^{0.126}$$ (15)

Interestingly, this expression indicates that *V*_*m*_ increases only slightly, and certainly less than linearly, with size. This probably holds true for all sea turtles species as, for simple scaling reasons, the value of *c* should always be close to 3 while the value of *b* proves to vary little between sea turtle species [23], remaining close to the generic value obtained for all reptiles (*b* = 0.83) [25].This is consistent with the results of Abecassis et al. [19] who show that, in juvenile loggerheads, *V*_*m*_/*L* (i.e. the maximum sustainable speed expressed in body lengths per second) decreases markedly with size.

The calibration of the movement model finally requires the specification of *v*_0_, the velocity scaling factor in Eq (15) and α, the factor controlling the concentration parameter in Eq (4). To estimate α, we simply hypothesize that, in the presence of a clear habitat gradient, an individual will systematically move into the half plane towards which **∇*h*** points, that is in a direction that does not differ by more than 90° from the optimal direction given by **∇*h***. In practice, we decide that a clear habitat gradient exists whenever the norm of the habitat gradient is larger than its median value*∇h*_*m*_. Since κ = 4 is the value of the heading concentration parameter of the von Mises distribution for which the probability of selecting movement directions deviating by more than 90° from the mean (optimal) direction becomes vanishingly small (see Fig 1), Eq (4) immediately yields α*∇h*_*m*_ = 4. Our simulations (see below) show that *∇h*_*m*_ is close to 1.3 10^−6^. We therefore choose α = 3 10^6^.

In the absence of speed measurements in juvenile leatherbacks, the calibration of *ν*_0_ can only rely on velocities measured in tracked adults. Based on growth curve Eq (13), simulated adults reach an SCL close to 1.4 m for which Eq (15) yields *V*_*m*_ ≈ *ν*_0_ and Eq (1) accordingly implies:

$$V_{s}\approx v_{0}\;\left(1-h\right)$$ (16)

This shows that *v*_0_ could readily be determined using either measurements of the maximum sustainable speed of adult leatherbacks or joint measurements of their swimming speed and habitat suitability. Unfortunately, a review of the literature on leatherback tracking experiments did not allow us to obtain such measurements. The most commonly reported speed estimate actually is the average speed, that is the mean speed computed over the complete trajectories of all turtles tracked in an experiment. Such average speeds typically range between 0.5 and 0.7 m/s [26–28]. Assuming that these average speeds are typical of an average habitat value (*h* = 0.5), Eq (16) indicates that *v*_0_ shall be between 1 and 1.4 m/s. We accordingly select *v*_0_ = 1.2 m/s. Simulation results (see below) show that with this choice of *v*_0_, the simulated swimming velocities of less than 3-year old individuals range between 0 and 0.3 m/s, while over 10-year old individuals have swimming velocities varying typically between 0.2 and 0.8 m/s, their mean speed being close to 0.6 m/s.

#### Thermal habitat calibration

The thermal habitat suitability index Eq (12) is characterized by 4 pivotal temperatures (*T*_*1*_ to *T*_*4*_) that define the range of water temperatures within which the modeled sea turtle species can maintain suitable body temperatures. Determination of the range of suitable body temperatures is thus requested before pivotal water temperatures can be specified.

Body temperatures measured in adult leatherbacks are typically in the range 24 to 28°C for individuals foraging in cold high-latitude areas [29,30] and 28 to 31°C for individuals tracked in the tropics [26,31]. Similar measurements are largely missing for hatchlings and juveniles. However, since hatchlings have a very small thermal inertia and little peripheral insulation, their body temperature has to be very close to the temperature of the water in which they are swimming. Hughes [32] analyzed ocean temperatures offshore several nesting beaches and concluded that leatherback hatchlings usually encounter waters between 25 and 31°C. A somewhat lower temperature of 24°C is also certainly suitable as it is the water temperature in which Jones et al. [24] successfully raised several leatherback hatchlings. We thus conclude that leatherbacks of all ages commonly experience body temperatures between 24 and 31°C. But does this range of observed body temperatures cover the whole range of suitable temperatures? The lowest suitable body temperature might actually be somewhat lower than 24°C, but not much. Indeed, the coagulation efficiency of leatherback’s blood decreases dramatically around 23°C [33] which suggests that such a low body temperature is not normally experienced [33,34]. We will accordingly assume that the lowest body temperature suitable for leatherbacks of all ages is 24°C.

On the contrary, the maximum suitable body temperature likely is well above 31°C: body temperatures reported in nesting females typically range between and 30.5 and 34.5°C [26,35–37] and hatchlings have an observed critical thermal maximum as high as 40.2°C [38], probably like adults [39]. The highest suitable body temperature shall thus be around 35°C or above. Such a high body temperature might be reached in the course of nesting activities but is probably never experienced at sea where water temperatures rarely exceed 30°C.

Assuming that leatherbacks never encounter unsuitably warm waters so that the condition *T*_*w*_ > *T*_*3*_ is never met in Eq (12), we can just skip the specification of the upper pivotal water temperatures *T*_*3*_ and *T*_*4*_. The specification of *T*_*1*_ and *T*_*2*_, the lower pivotal water temperatures, is less simple. Indeed, thanks to various morphological, physiological and behavioral adaptations, leatherbacks are able to maintain *T*_*b*_ well above *T*_*w*_ [22,29,30,40,41] and the temperature gradient (*T*_*b*_ -*T*_*w*_) that they can maintain proves to increase with their mass [40] and level of activity [41]. The evolution of this gradient is actually governed by the heat budget of the concerned individual. Based on the steady-state version of this budget, Bostrom et al. [41] deduce that, for a leatherback of mass *M*:

$$\;T_{b}-T_{w}=d\;M{\;}^{0.5}$$ (17)

where *d* is a coefficient directly proportional to the individual’s metabolic heat production. The evolution of *d* with the metabolic rate (*MR*) can be made more explicit by rewriting Eq (17) under the form:

$$T_{b}-T_{w}=d_{0}\;\left(MR/RMR\right){\text{ M }}^{0.5}$$ (18)

where *d*_0_ is the value of *d* for a resting individual. Based on observations of quiescent captive juveniles, Bostrom et al. [41] estimated *d*_0_ = 0.21. As 24°C is the assumed minimum suitable body temperature for leatherbacks of all ages/mass, Eq (18) implies that this value of *T*_*b*_ is obtained for:

$$T_{w}=24-0.21\;\left(MR/RMR\right){\text{ M }}^{0.5}$$ (19)

This equation provides the basis for estimating *T*_*1*_ and *T*_*2*_. Indeed, *T*_*1*_ can be defined as the strictly minimum water temperature within which a most active individual is able to maintain *T*_*b*_ = 24°C while *T*_*2*_ is the water temperature within which a resting individual will effortlessly obtain this same body temperature. Therefore:

$$T_{2}=24-0.21\;M^{0.5}$$ (20)

Then, the estimation of *T*_*1*_ depends on the maximum value of the *MR*/*RMR* ratio that a leatherback can sustain. We expect this maximum value to be somewhere between 4 and 5 as nesting is often quoted as leatherbacks’ most strenuous activity, and metabolic rates measured in nesting females reach 4 to 5 times their RMR [40,41]. Such an estimate is consistent with the results of Peterson et al. [42] who analyzed the sustained metabolic rate (*SMR*) in a number of free ranging vertebrates. They observed that the *SMR*/*RMR* ratio is always smaller than 7 and, in most case, smaller than 5. In particular, this ratio remains below 5 in all surveyed ectothermic species (lizards).

We thus conservatively estimate that leatherback turtles can sustain metabolic rates up to 5 times their RMR so that the minimum water temperature (*T*_1_) in which a (very) active individual will be able to maintain a minimum body temperature of 24°C is:

$$T_{1}=24-1.05\;M^{0.5}$$ (21)

Using Eqs (13) and (14) to express mass as a function of age, *T*_*1*_ *and T*_*2*_ can then readily be expressed as a function of age as needed:

$$T_{1}=24-18.56\;\left[1-{\text{e}}^{-0.226\left(a+0.17\right)}\;\right]{\;}^{1.43}$$ (22)

$$T_{2}=24-3.71\;\left[1-{\text{e}}^{-0.226\left(a+0.17\right)}\;\right]{\;}^{1.43}$$ (23)

**Fig 3:**
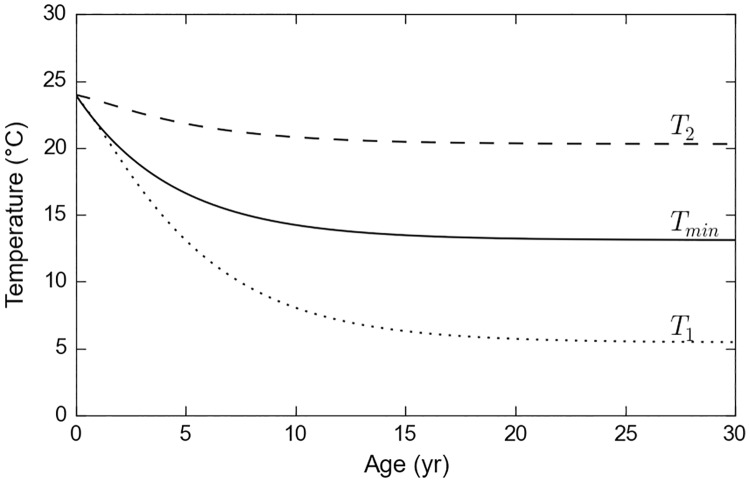
Evolution with age of the lower pivotal water temperatures *T*_*1*_ and*T*_*2*_, and the minimum tolerated temperature (*T*_*min*_) defined by GAL.

It is worth noting that the minimum tolerated temperature (*T*_*min*_) empirically determined by GAL for leatherbacks fits right in-between these two pivotal temperatures (Fig 3).

**Fig 4:**
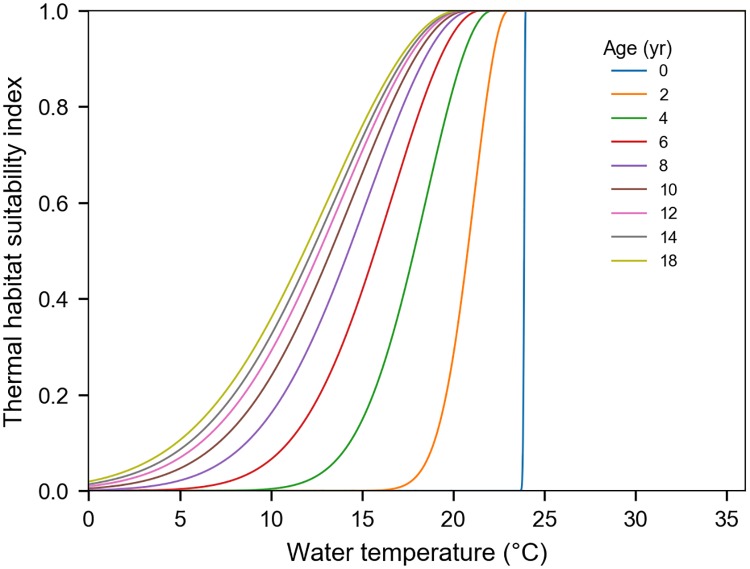
Thermal habitat suitability index as a function of water temperature for leatherbacks of different ages.

We have now completed the parameterization of the thermal habitat suitability index. This index is shown in Fig 4 for leatherbacks of various ages. As expected for hatchlings, this index falls almost immediately down to zero in water temperatures below 24°C. For larger/older individuals, markedly colder waters remain suitable, but the suitability index becomes very small so that modeled individuals will tend to move out of these cold waters.

#### Feeding habitat calibration

The definition of the feeding habitat suitability index Eq (11) is based on the local prey density (*P*) and the individual rate of food consumption *F*(*a*). In the absence of synoptic estimates of the distribution of jellyfish, the main diet of leatherbacks, we follow Saba et al [43] and use satellite-derived estimates of the net primary production (NPP) as a proxy for *P*.

**Fig 5:**
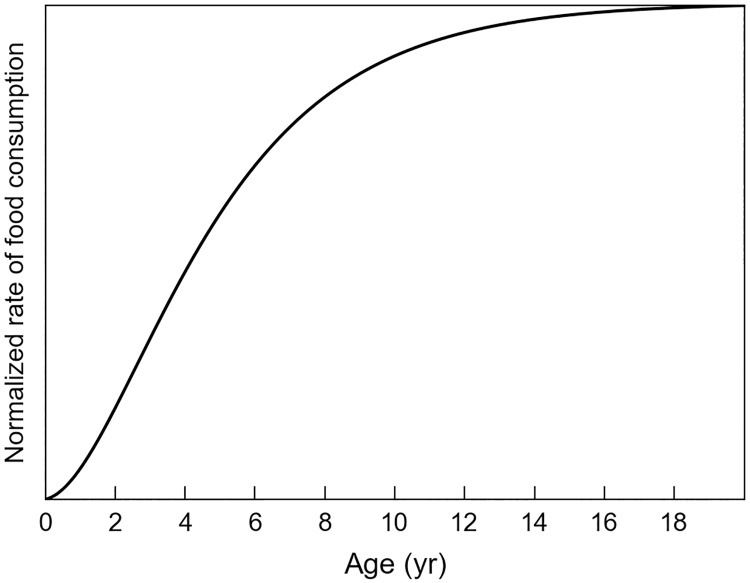
Normalized rate of food consumption *F*_*0*_(*a*)as a function of age.

The rate of leatherback’s food consumption has been estimated by Jones et al. [44]. This estimate is expressed in kilograms of jellyfish consumed per year and must thus be rescaled to account for the fact that NPP is used as a proxy for *P* instead of jellyfish density. To do so, we first define *F*_*0*_(*a*), a normalized and nondimensionalised version of the Jones et al [44] estimate of the rate of food consumption:

$$F_{0}\left(a\right)=f_{0}\;\frac{x\;{\left(1-x\right)}^{1.86}}{1-\;{\left(1-x\right)}^{0.094}}\text{ with }x=\;e^{-0.299\left(a+0.17\right)}$$ (24)

where *f*_0_ = 0.094 so that *F*_*0*_(*a*)→1 when *a* →+∞ (Fig 5).

The actual rate of food consumption is then given by:

$$F\left(a\right)=P_{0}\;F_{0}\left(a\right)$$ (25)

where *P*_0_ is a scaling factor expressed in the same units as *P*, the prey field or its proxy (in this case, NPP). The feeding habitat suitability index Eq (11) thus finally reads:

$$h_{F}\;\left(x,y,t,a\right)=Min\left\{1,\;NPP\left(x,y,t\right)/\left[P_{0}\;F_{0}\left(a\right)\right]\right\}$$ (26)

This expression thus clearly shows that *P*_0_ is the threshold value of *NPP* above which the adults’ habitat suitability reaches its maximum value (*h*_*F*_ = 1). Estimation of that parameter is probably the most uncertain part of the model’s calibration. One could simply decide that *h*_*F*_ = 1 for the maximum NPP value encountered in the North Pacific. However, this would yield a very high *P*_0_ value, only encountered in the richest coastal areas where the accuracy of satellite-derived NPP estimates is, unfortunately, the weakest [45]. Furthermore, known favorable pelagic foraging areas (such as the North Pacific Transition Zone) have much weaker NPP levels than most productive coastal areas. They would thus only have a weak suitability index if *P*_0_ was set at a high coastal NPP value. We therefore set the value of *P*_0_ at the level corresponding to the 90^th^ percentile of the NPP distribution in the North-Pacific. With this choice, *P*_0_ = 55 mmol C m^2^ day^-1^. This is well below the high NPP values measured in coastal regions and typically in the range of the NPP values found in productive pelagic areas of the North Pacific [46].

### Model simulations

GAL previously investigated the passive dispersal of juveniles from the western Pacific leatherback population nesting in New Guinea. They simulated 6-year long passive drift trajectories of hatchlings emerging from (a) Jamursba-Medi, the major nesting beach of this population situated on Bird’s Head Peninsula, and (b) Kamiali, a less used nesting beach in the Huon Gulf [47]. They identified several dispersal pathways from these beaches into the North and South Pacific Ocean, the Indonesians seas and the Indian Ocean. As a first test for STAMM, we will focus here on the main nesting beach of Jamursba-Medi and more specifically on the dispersal from this beach into the North Pacific Ocean. This is indeed a pathway along which large habitat variations, and hence significant habitat-driven movements, are expected.

### Technical set up

The technical setup of our simulations is similar to that of GAL. In particular, we use the same surface current data, the same trajectory simulation software, the same daily time step and the same hatchlings release procedure.

Surface currents vectors (***V***_***c***_) are taken from daily outputs of the GLORYS-1 (G1) reanalysis of the World Ocean circulation [48] performed by the Mercator-Ocean centre (www.mercator-ocean.fr) with the NEMO numerical ocean model (www.nemo-ocean.eu). The G1 model has an eddy-permitting horizontal resolution of 0.25° and 50 vertical layers. It covers the 7-year period going from 01/01/2002 to 31/12/2008. The G1 reanalysis assimilates satellite-derived sea level anomalies and sea surface temperature (SST) data as well as in situ temperature and salinity measurements. It provides a close-to-reality simulation of the World Ocean dynamics that proves to be specially well suited for simulating surface drifter trajectories [49].

Trajectories are simulated using the ARIANE Lagrangian trajectory simulation software (www.univ-brest.fr/lpo/ariane). This program uses an accurate quasi-analytical solution of the advection equation [50]. To produce passive drift trajectories, GAL fed ARIANE with daily surface currents (***V***_***c***_) provided by G1. In STAMM, we feed ARIANE with turtles’ velocities on the ground (***V***_***g***_ = ***V***_***c***_ +***V***_***s***_) and thus obtain trajectories resulting from the combined effect of current drift and active swimming. Surface current values are used as diving is not explicitly simulated here. Parameterization of the diving activity might be envisioned in subsequent versions of STAMM. In that case, currents at different depths shall be taken into account.

To simulate the effect of the swimming frenzy, hatchings are released offshore, in a 0.25° x 0.25° area centered about 40 km off Jamursba-Medi. The release positions are uniformly distributed inside this area. The nesting season at Jamursba-Medi extends from April to September, peaking in July [51]. Taking into account a 2-month incubation period, simulated hatchlings are released between June and November. The number of releases per day fits a truncated normal distribution that peaks in mid-September, exactly as in GAL.

### Estimation of the habitat suitability index and its gradient

The thermal habitat suitability index Eq (12) is computed using the water temperature simulated in the first layer (0 to 1 m) of the G1 reanalysis. This temperature is given at the center of each grid cell and is close to the satellite-derived SST assimilated into G1. For feeding habitat determination, NPP is obtained from the Ocean Productivity web site (www.science.oregonstate.edu/ocean.productivity/), using the VGPM algorithm [52]. This NPP estimate is available for the entire G1 period (2002 to 2008) with a spatial resolution of 1/6° and a temporal resolution of 8 days. Simple linear interpolation in time and bilinear interpolation in space is used to estimate daily values of the NPP at the center of each G1 grid cell. Habitat gradients are then computed at the center of these grid cells using simple centered finite differences. Daily values of the habitat suitability and their gradients are then used to produce daily swimming speeds.

### Simulation period and number of particles

STAMM is designed to simulate the swimming activity of juvenile sea turtles motivated by the search for food and suitable water temperatures. In leatherbacks, such a model shall be valid from the end of the swimming frenzy until the onset of the first reproductive migration. The age at which leatherbacks reach sexual maturity is still largely uncertain. Published estimates range from a few years up to nearly 30 years but most results situate sexual maturity between 12 and 18 [53]. We therefore chose to run STAMM over a 18-year-long period which likely covers the complete pelagic juvenile stage. For comparison purposes, we also ran an 18-year long passive simulation which is simply an extended version of the 6-year-long passive drift simulation of GAL. For the sake of simplicity we will refer to this passive-drift simulations and the one performed with STAMM as the passive dispersal and active dispersal simulations respectively. Similarly, simulated individuals will be referred to as passive and active turtles.

To perform 18-year-long simulations with 7-year-long forcing data sets (G1 and NPP), we simply loop the forcing fields. This means that the simulations continue after December 2008 using again data starting in January 2002. This process is repeated until the last released turtle reaches the age of 18.

All simulations involve 5000 particles (simulated turtles) released off Jamursba-Medi between June and November 2002. Note that out of the 5000 simulated turtles, roughly 3000 (passive and active) individuals finally circulate in the North Pacific while the others drift into the Indonesian seas, the Indian Ocean or the South Pacific. Only the dispersal into the North Pacific is analyzed here.

## Results and discussion

**Fig 6:**
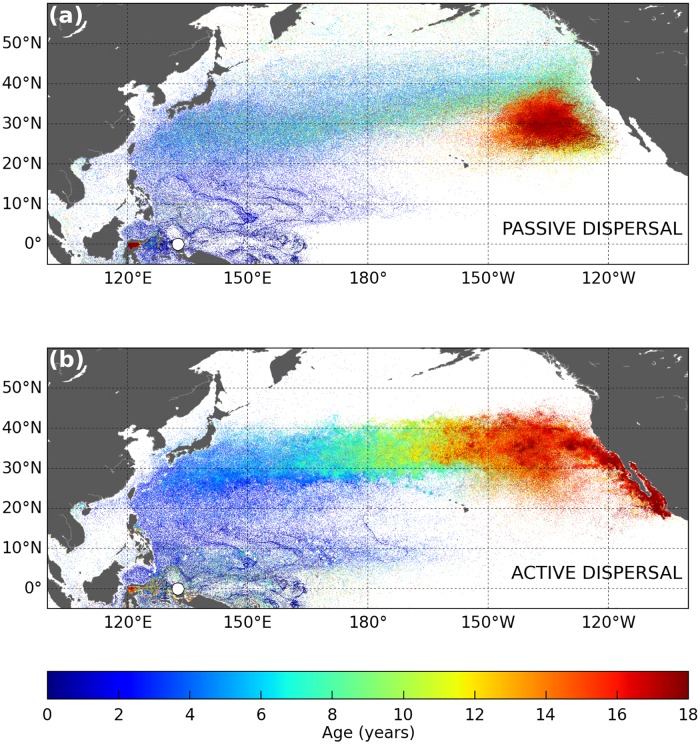
Simulated 18-year long trajectories of (a) passive and (b) active leatherbacks dispersing in the North Pacific. Individuals are released offshore Jamursba-Medi nesting beach (white dot on the map). The color along each track evolves with the age of the simulated turtle.

The simulated 18-year long trajectories of active and passive turtles dispersing from Jamursba-Medi into the North Pacific are shown in Fig 6. An animation of their evolution with time is provided as supporting information (S1 and S2 Figs).

### Similarities between passive and active dispersal patterns

**Fig 7:**
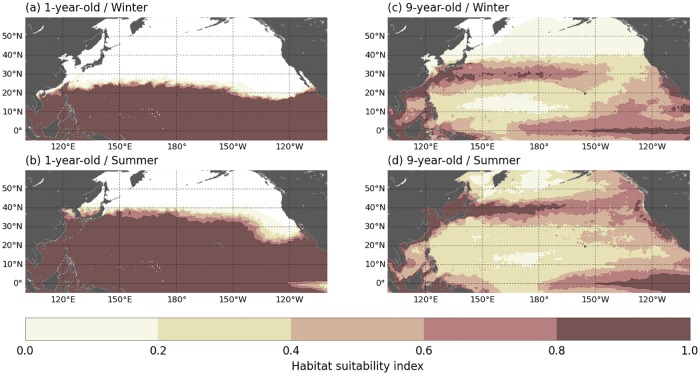
Maps of habitat suitability index. Maps for 1- and 9-year-old leatherbacks during winter (January to March) and summer (July to September).

Our results (Fig 6) show that the simulated active and passive spatial dispersal patterns are broadly similar and clearly shaped by the ocean currents. During their two first years of life, simulated juveniles disperse mostly into the western tropical Pacific Ocean, at latitudes below 25°N. In this area, the similarity between the active and passive dispersal simulations is striking and indicates that the simulated swimming velocities are very weak compared to the current velocities. This occurs because (a) water temperatures in the dispersal area never fall below *T*_*2*_ (except near 25°N, in wintertime) and (b) food requirements of young individuals are small and easily met in the visited areas. Therefore *h* ≈ 1 (Fig 7a and 7b) and thus *V*_*s*_ ≈ 0.

**Fig 8:**
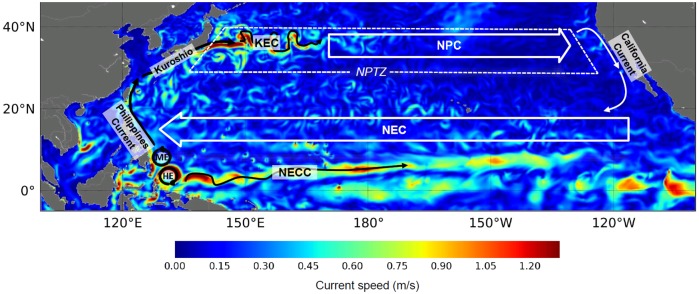
Schematic map of surface currents in the North Pacific. NECC: North Equatorial Countercurrent; NEC: North Equatorial Current; HE Halmahera Eddy; ME: Mindanao Eddy; KEC: Kuroshio Extension Current; NPC: North Pacific Current; NPTZ: North Pacific Transition Zone. Underlying current speeds are those of the G1 ocean reanalysis on October 1^st^, 2002.

During their two first years, the Jamursba-Medi hatchlings entrained into the North Pacific, first circulate around the anti-cyclonic Halmahera eddy (Fig 8) and can then follow two main pathways. Some individuals leave the Halmahera eddy to circulate anti-clockwise around the Mindanao eddy and then enter the Philippines current before reaching the powerful Kuroshio which rapidly entrains them northward, towards Japan. This pathway will be referred to as the Kuroshio pathway. Hatchlings following the second pathway leave the Halmahera eddy to flow eastward directly into the North Equatorial Counter-Current (NECC) until shear-induced lateral mixing entrains them into the North Equatorial Current (NEC), that is back westward into the clockwise circulation of the North Pacific subtropical gyre. Drift into the NECC can be very brief or extend far into the Central Pacific, sometimes as far as 170°W. All NECC individuals finally recirculate inside the subtropical gyre and reach the North Pacific Transition Zone (NPTZ) in a quite scattered way, generally well offshore Japan. The NPTZ is the broad frontal area separating the warm subtropical gyre from the colder, but more productive subpolar gyre. It is situated typically between 30 and 40°N.

At the end of their second year of life, most passive and active turtles have reached middle-latitude areas. The Kuroshio individuals are found off Japan, close to 30°N. The NECC individuals are more scattered, but generally positioned further east and at somewhat lower latitudes. Both groups then keep moving broadly northward, rapidly for the Kuroshio individuals and more slowly for the NECC individuals. As they progressively approach 35°N their drift motion becomes predominantly eastward, following first the Kuroshio extension current and then the North Pacific current. After several years, both the active and passive individuals finally reach (or nearly so) the west coast of North America.

This broad similarity between the simulated active and passive dispersal patterns clearly shows that the ocean circulation is the main factor governing broad-scale dispersal in juvenile sea turtles. This likely is the main reason why the passive drift hypothesis has been so successful at explaining the large-scale distribution of oceanic juveniles in many sea turtle populations [10–12,54].

However, beyond this apparent similarity, a closer examination of our results reveals significant differences between the passive and active dispersal scenarios. Active turtles do not disperse as far north as passive turtles. They undertake seasonal migrations and cross the Pacific more slowly. Then, having crossed the Pacific, active turtles tend to concentrate along the coast of California and Baja California while passive turtles remain somewhat offshore. These differences have important consequences on the vital rates, spatial distribution, individual fitness, likelihood of interactions with fisheries, and thus whole population dynamics. These are analyzed below.

### Differences between passive and active dispersal scenarios

#### Seasonal migrations

Seasonal migrations are commonly observed in both juvenile and adult chelonid sea turtles, e.g. [55–57]. They are known to occur in sub-adult and adult leatherbacks [58,59] and are thus expected in juvenile leatherbacks. Such migrations cannot be generated by the passive drift mechanism but naturally appear when habitat-driven movements are added. The S2 animation clearly shows that, as soon as active turtles reach the middle latitudes, they undertake north-south migrations following the seasonal movements of the habitat suitability index. Two conditions need to be met to trigger a complete seasonal migration cycle. The first one is that juveniles need to have reached latitudes where wintertime water temperatures fall below the lower bound of their thermal preferendum (*T*_*2*_). In that case, the thermal habitat suitability gradient leads simulated individuals to retreat southward towards warmer waters. The second condition is that turtles must move back north as the water warms up during spring. This is achieved when simulated juveniles reach an age at which their food requirements are large enough to create a foraging habitat gradient that leads them towards richer foraging grounds, generally found northward.

The need to move south to escape cold waters first appears in the Kuroshio individuals, at the end of the second year of simulation (note that, at that time, these individuals are only 1-year old, as they were born between June and November of the first simulation year). Unfortunately many of these individuals appear to be unable to swim fast enough to avoid overly cold waters (see the discussion about mortality in the next section). The need to move north in search for better foraging grounds appears later, mostly during the fourth year of simulation, when seasonal migrations become fully visible in the S2 animation.

The NECC individuals remain longer in warmer and sufficiently productive waters and initiate seasonal migrations only when they reach the colder waters of the NPTZ. This typically happens when they are 3 up to 6-year old.

Simulated habitat-driven movements, and the resulting seasonal migrations, also clearly shape the northern boundary of the dispersal area. In the passive dispersal simulation, this boundary is very diffuse and situated roughly between 40 and 50°N (Fig 6a). In the active dispersal simulation, a more clear-cut boundary is observed around 40°N (Fig 6b). This is the latitude above which the water temperature falls below *T*_*2*_ towards the end of the fall and leads active turtles to undertake their southward wintertime migration.

#### Cold-induced mortality

**Fig 9:**
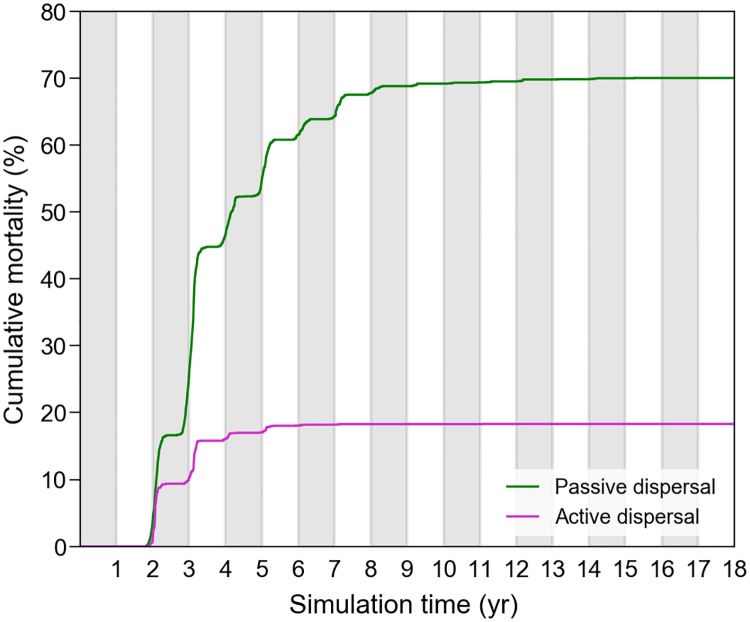
Cumulative cold-induced mortality in passive and active turtles.

The pivotal water temperature *T*_*1*_ being defined as the temperature below which a turtle cannot survive long, we will accordingly assume that a simulated turtle dies if it experiences *T*_*w*_ <*T*_*1*_ during 10 days or more. A simple analysis of the water temperatures encountered along the trajectories of all active and passive turtle thus immediately reveals when and where cold-induced mortality occurs. The very first deaths actually occur at the end of the second year of simulation (Fig 9), that is when the first (both active and passive) Kuroshio individuals arrive offshore Japan in wintertime. During that first winter spent at mid-latitudes, death is diagnosed for 16% of the passive individuals and 10% of the active ones. The difference between the passive and active cases is even larger during the next winter: another 29% of the passive individuals, but only 6% of the active ones, die from hypothermia. During the following winters, an additional 25% of the passive individuals, but only 3% of the active ones suffer cold-induced mortality. This indicates that, after about 3 years, most active individuals are sufficiently cold-resistant and powerful swimmers to efficiently exploit the rich waters found in the northern part of the NPTZ during summer and fall and then rapidly retreat southward as winter arrives and water temperature drops down. At the end of the simulation, the cumulative mortality reaches 70% in passive individuals but only 19.3% in active turtles. This clearly shows that simulated habitat-driven movements, and seasonal migration in particular, are very efficient at limiting, but not completely suppressing, cold-induced mortality.

**Fig 10:**
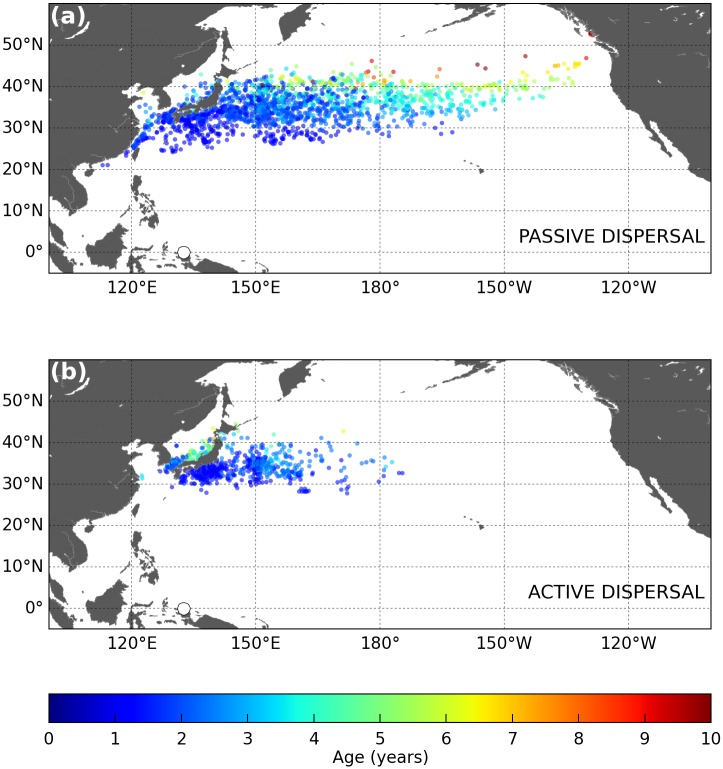
Spatial distribution of cold-induced death events in passive (a) and active (b) turtles.

Death events in passive turtles are widely dispersed in the North Pacific (Fig 10a). Young (*a* ≤ 3 yr) passive turtles die from hypothermia generally north of 25°N and west of the dateline. These are mostly Kuroshio individuals. Older, more cold-resistant, passive individuals are observed to die mostly above 35°N and east of the dateline. Death events in active turtles are much less dispersed. They mostly occur offshore Japan, in 2- to 3-year-old Kuroshio individuals. (Fig 10b). Somewhat older individuals are also observed to die further east offshore Japan and in the Sea of Japan.

**Fig 11:**
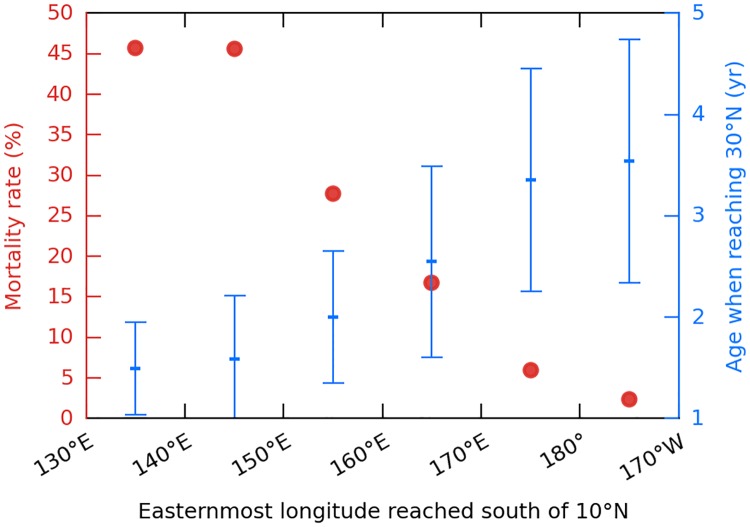
Link between cold-induced mortality and initial dispersal pathway. Cold-induced mortality rate and mean age (±1 std) when reaching 30°N in active turtles pooled as a function of the easternmost longitude reached in the NECC (south of 10°N).

The link between cold-induced mortality in active turtles and their initial dispersal pathway (Kuroshio or NECC) is easily highlighted by analyzing their mortality as a function of the easternmost longitude reached south of 10°N, that is when simulated turtles (possibly) circulate into the NECC. Turtles that do not drift further east than 140°E have a mortality rate approaching 50% (Fig 11). These are the Kuroshio individuals plus some individuals that circulate only briefly into the NECC. Turtles that circulate further east into the NECC have much lower mortality rates, well below 10% for the individuals drifting east of 170°E. This decrease in the mortality rate is clearly related to the age at which individuals enter the colder mid-latitude waters. Kuroshio individuals reach 30°N at a mean age between one and two. Individuals that drift past 170°E are typically 3- to 4-year-old when they reach that latitude. By then they are more cold-resistant and sufficiently powerful swimmers to escape overly cold waters when needed.

#### North Pacific crossing time

GAL defined the North Pacific crossing time (PCT) as the age at which simulated individuals first reach the longitude of 140°W and showed that passive turtles emerging from Jamursba-Medi can reach that longitude within 5 to 6 years. However, in doing so, all of them encounter water temperatures below their minimum tolerated temperature (*T*_*min*_) and thus likely die. Furthermore GAL noted that the presence of 5 to 6-year old leatherbacks in the eastern North Pacific is unlikely as all leatherbacks incidentally caught east of 150°W by the Hawaii-based longline fleet are large individuals (SCL>1.3m), likely older than 10 years. GAL thus concluded that (a) Jamursba-Medi hatchlings are unlikely to achieve a fast, purely passive, crossing of the North Pacific Ocean and (b) a slower crossing, lasting over 10 years or more, would be more consistent with observations. GAL then argued that an active dispersal scenario involving active seasonal north-south migrations could generate such a slower crossing of the North Pacific basin. Indeed, individuals retreating each winter towards the center of the subtropical gyre would encounter weaker eastward currents and would thus cross the North Pacific basin more slowly. A detailed analysis of our passive and active simulations actually reveals that, besides the weaker eastward currents encountered by seasonally-migrating individuals, another more important mechanism explains why active turtles likely cross the Pacific more slowly than the passive ones.

**Fig 12:**
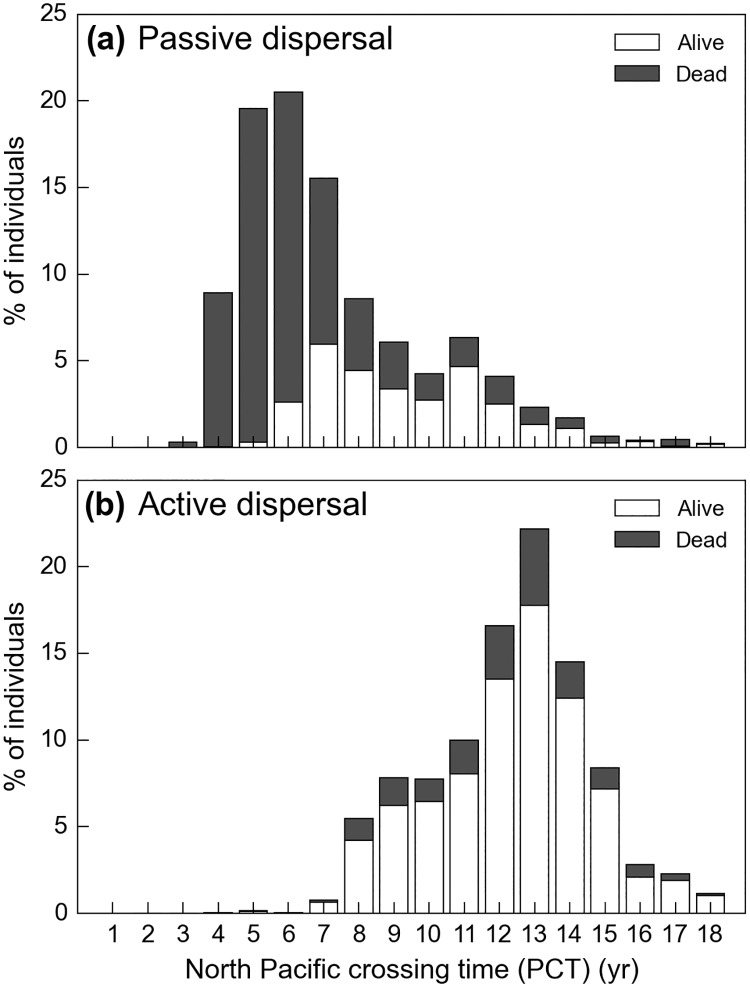
Histogram of the North Pacific crossing times (PCT) for (a) passive and (b) active turtles.

Using the PCT definition of GAL, we observe that the number of individuals reaching the longitude of 140°W indeed peaks at 5–6 years for passive turtles and much later (12–13 years) for active turtles (Fig 12). In addition, passive turtles with a short PCT prove to suffer massive cold-induced mortality, as previously observed by GAL. Actually, all passive turtles with a PCT shorter than 6 years die in the GAL simulation while a few of them survive in our passive dispersal simulation. This is simply due to the fact that our cold-induced mortality criterion is less stringent than the one used by GAL (i.e. *T*_*1*_ < *T*_*min*_, see Fig 3).

Interestingly also, we observe that about one third of our passive turtles cross the Pacific in more than 6 years and have a significantly lower mortality rate than those with a shorter PCT. On the contrary, the mortality rate of active turtles bears no apparent correlation with the PCT. This difference in the mortality rates is easily understood looking at the speed at which passive and active turtles with different PCT move towards higher latitudes and thus encounter cold waters.

**Fig 13:**
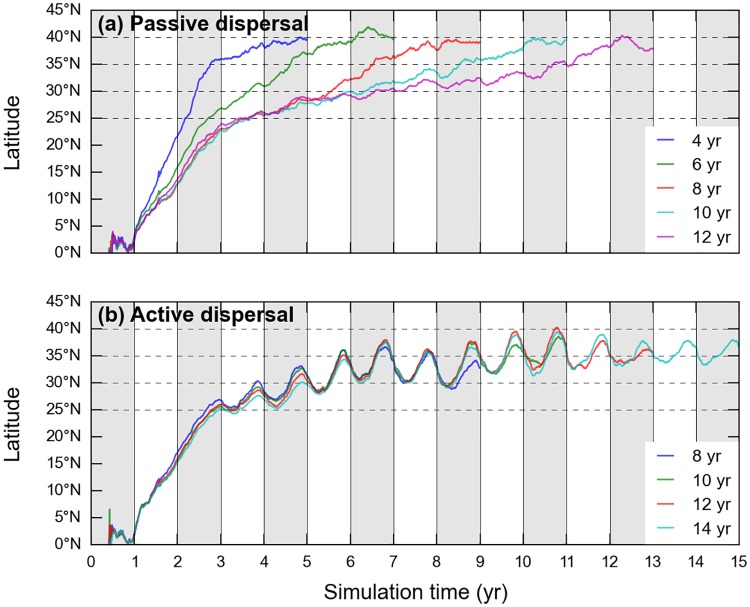
Mean latitude as a function of simulation time for groups of (a) passive and (b) active turtles with the same PCT. A group with a crossing time of N years is actually made of all turtles having a crossing time between N years– 6 months and N years + 6 months.

Passive turtles with different PCT display markedly different trajectories (Fig 13a). Those with the shortest crossing time are clearly Kuroshio individuals: they have the fastest northward progression and hence the highest cold-induced mortality rate. They reach 35°N, the mean latitude of the Kuroshio, within 2 years and then circulate rapidly into this mighty eastward current towards the other side of the Pacific. Passive turtles with a longer crossing time progress more slowly towards North and are thus more cold-resistant when entering cold mid-latitude waters. Their crossing time is longer mostly because they reach 35°N later (and more to the East) than the Kuroshio individuals. They thus encounter weaker eastward currents and therefore take longer to cross the Pacific.

On the contrary, groups of active turtles with different crossing times prove to have very similar trajectories (Fig 13b). They thus visit similar latitudes at similar times and accordingly have similar cold-induced mortality rates.

We finally have to explain why the PCT of active turtles is, on average, much longer than that of passive turtles. To do so, let us concentrate on the two most numerous groups: the group of passive turtles with a PCT of 6 years and the group of active turtles with a PCT of 13 years. As the swimming velocities are initially very small, the mean trajectories of the active and passive groups remain close to each other during their two first years of life. At the end of this period, the mean positions of the two groups are close to (25°N, 148°E). From there, both groups have to cover a distance towards East of about 6600 km before reaching 140°W. The passive group does that in 4 years, the active one in 11 years. To cover that distance, the passive group is pushed by currents having a mean zonal (eastward) component *u*_*c*_ = 0.052 m/s. As expected, this is almost exactly the speed needed to cover 6600km in 4 years. For the reason explained by GAL, the active group experiences a weaker mean eastward current speed: *u*_*c*_ = 0.039 m/s. At such a speed however, this group would cover the distance of 6600 km in 5.4 years and would thus have a PCT of 7.4 years, not 11 years! The missing part of the puzzle is that active turtles actually swim (on average) towards west, at a mean zonal speed *u*_*s*_ = -0.02 m/s. Their zonal speed on the ground (*u*_*g*_ = *u*_*c*_ + *u*_*s*_) is thus only 0.019 m/s, which is indeed the speed needed to cover 6600 km in 11 years.

**Fig 14:**
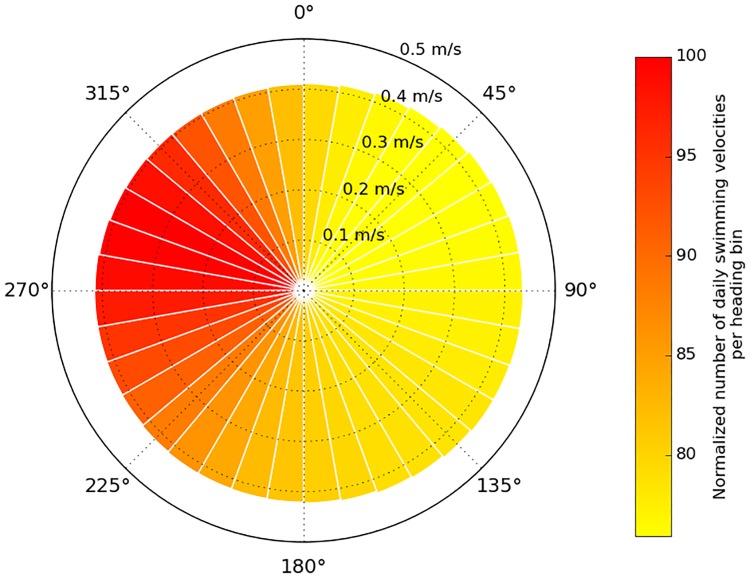
Circular histogram of the daily swimming velocities. This histogram includes the daily swimming velocities of all active turtles found between 120°E and 140°W and north of 25°N. The color in each 10°-wide heading bin reflects the number of daily swimming velocities found in this bin. This number is normalized so that the most populated bin has an index of 100.

This, possibly surprising, result must be properly interpreted: that active turtles have a mean westward swimming speed of -0.02 m/s does not imply that they permanently swim against the dominant eastward flow. They actually swim faster (daily mean *V*_*s*_ = 0.42±0.23 m/s) but in all directions (Fig 14). The distribution of their heading angles however is clearly skewed towards west so that the resulting mean value of *u*_*s*_ is negative (i.e. westward). The origin of this westward bias in swimming velocities is analyzed in the next section.

#### Habitat gradients and swimming speeds

**Fig 15:**
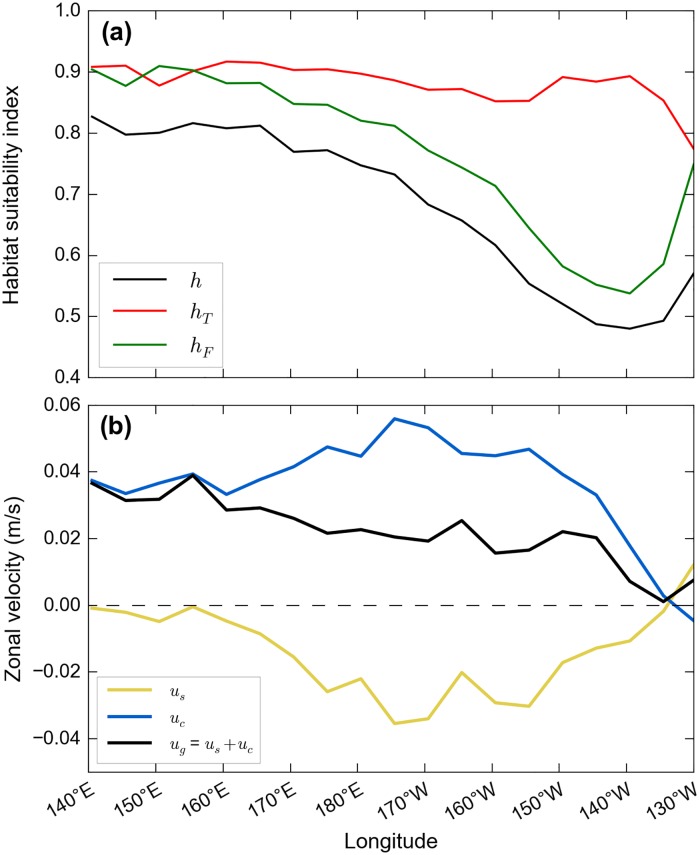
Zonal variations in habitats and speeds. Averaged longitudinal variations of (a) habitat suitability indices (*h*, *h*_*T*_, *h*_*F*_) and (b) zonal speeds (*u*_*s*_, *u*_*c*_, *u*_*g*_) along the trajectories of active turtles crossing the North Pacific.

Simulated habitat-driven swimming velocities being directed towards more favorable habitats, these velocities shall be westward if better habitats are found to the west. This is indeed the case in the 30-to-40°N latitude band within which active turtles cross the Pacific. Productivity triggered by the highly turbulent Kuroshio extension current is specially high offshore Japan and more generally in the western part of the NPTZ (Fig 7c and 7d). Active turtles crossing the Pacific Ocean thus encounter favorable feeding conditions in the western part of the basin before moving through progressively poorer areas of the central and eastern Pacific. Fig 15a confirms that the foraging habitat suitability index (*h*_*F*_) is maximum to the west of the basin, decreases steadily through the central and eastern Pacific and finally increases again when active turtles reach the productive California current region. The total habitat suitability index (*h*) displays similar longitudinal variations as the thermal habitat suitability index (*h*_*T*_) proves to vary little with longitude, except off California where colder waters induce a decrease in *h*_*T*_). In agreement with these zonal variations of *h* and its gradient, the mean value of *u*_*s*_ remains close to zero in the westernmost part of the basin (Fig 15b). It becomes clearly negative in a broad longitude band between 170°E and 150°W and then comes back to zero near 135°W, before finally changing sign when the zonal habitat gradient also changes sign and leads active turtles to swim towards the rich Californian feeding grounds.

#### Spatial density distribution

The spatial density distribution of juvenile sea turtles is a critically needed information to locate high use areas and evaluate risks of interactions with fisheries. A simple way of obtaining density maps from individual tracking experiments is to bin daily positions (or “turtle days”) in regular boxes and then use the number of turtle days per box as an estimate of the turtle density [60,61]. This is actually only a proxy of the turtle density as the number of turtle days per box increases not only with the local density of turtles but also with the residence time of each turtle in the box.

**Fig 16:**
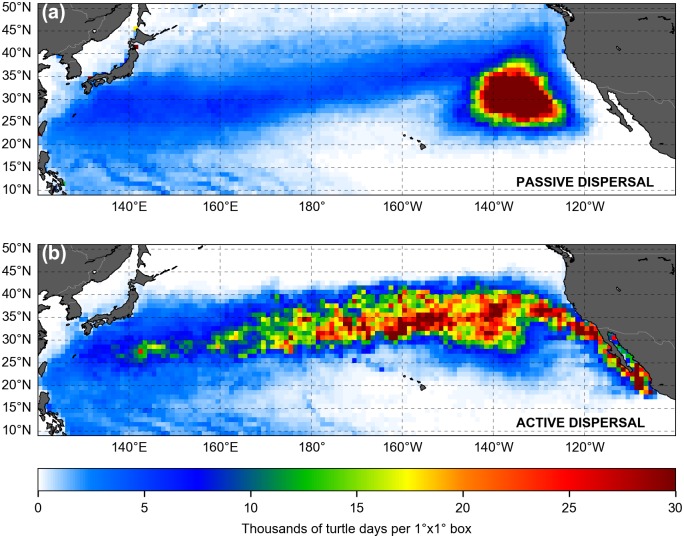
Number of turtle days in 1°x1° boxes for (a) the passive and (b) the active dispersal simulations.

Using the daily positions generated by our passive and active dispersal simulations, the number of turtle days per 1°x1° boxes is easily computed. Even if the passive and active dispersal patterns are broadly similar (Fig 6), the corresponding density maps are strikingly different (Fig 16). Pushed by (on average) faster eastward currents and not forced to converge towards favorable habitats, passive turtles disperse widely in the North Pacific and rapidly cross it. Low turtle densities are thus recorded almost everywhere in the basin, except in the easternmost part of the subtropical gyre where the convergence of Ekman currents tend to aggregate passive turtles, just like passive plastic debris [60]. On the contrary, active turtles target favorable habitats and follow them seasonally. They thus gather in the same latitude band in the same season. This latitude band oscillates typically between 25 and 40°N (see Fig 13b) so that the turtle density is especially high between these two latitudes. In that latitude band also, the density clearly increases from west to east. The relatively low densities in the western part of the basin are due to the fact that simulated individuals are still widely dispersed in latitude. NECC individuals only progressively reach 25°N, sometimes at longitudes as far east as 170 to 180°E. However, once most individual are gathered in the 25 to 40°N latitude band, the number of turtle days keeps increasing towards East. This is mostly due to the fact the mean zonal velocity on the ground (*u*_*g*_) decreases nearly steadily towards east (Fig 15b) and causes simulated turtles to stay longer in each longitude box. The density distribution finally changes shape at about 135°W, where the mean swimming speed changes direction and becomes westward. From there active turtles start spreading along the coast of California and Baja California.

Although no juvenile leatherback tracking data are available to validate the simulated density map, we note that Briscoe et al. [61] recently produced a comparable map for juvenile loggerheads tracked in the North Pacific. Interestingly, their density distribution displays some similarity with ours. In particular, loggerhead trajectories are also concentrated in the 30 to 40°N latitude band and residence times are especially high in the central part of the basin (180°W to 160°W).

#### Food availability, growth and energy accumulation

**Fig 17:**
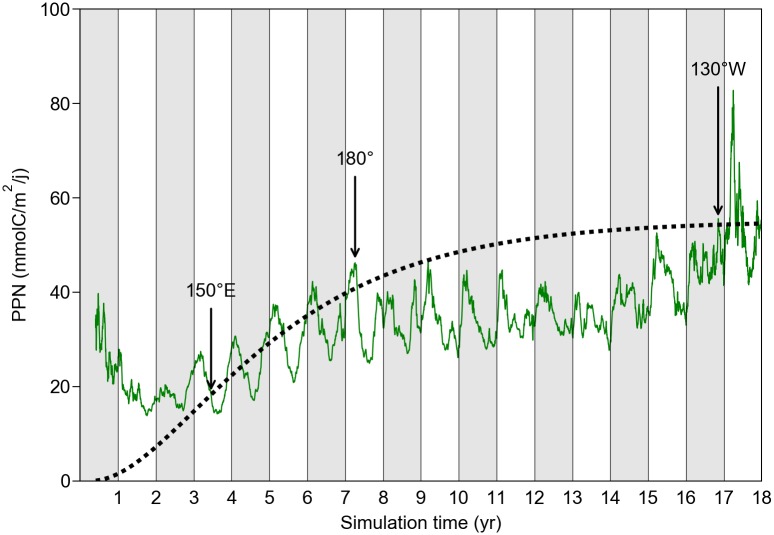
Mean value of the NPP encountered by active turtles crossing the Pacific alive. The food requirement curve *F*(*a*) is that of individuals born at the peak of the emergence period, that is on September 15 of the first simulated year. Tags with the mean longitude of the simulated turtles are inserted at various simulation times to establish the link between the position and simulation time.

As discussed in the previous section, active turtles initiate their crossing of the Pacific in the productive waters found in the western part of the NPTZ. They then transit through the less-productive central and eastern North Pacific before finally entering the rich waters of the California current system. Model results accordingly indicate that the NPP encountered by active turtles during their 3 first years of life is, on average, more than sufficient to meet their daily food requirements, i.e. NPP > *F*(*a*) (Fig 17). During the next 4 year, that is when active turtles move between 150°W and 180°, NPP is only marginally (seasonally) sufficient. NPP then becomes insufficient to fully meet the food requirements of the simulated individuals crossing the eastern part of the basin. Slower growth and increased starvation-induced mortality is thus expected in this area. Finally, NPP increases markedly as active turtles get into the California current region (at about 130°W) where high productivity allows larger/older individuals to fulfill their food requirements. This likely signals the beginning of a period of energy accumulation after which the first reproductive migration might occur. If this was the case, sexual maturity would be reached after about 15 years, the mean time needed for active turtles to reach 130°W.

A similar analysis of the NPP encountered by passive turtles shows that (a) passive turtles which cross the Pacific without encountering lethal temperatures experience generally lower NPP values than active turtles and (b) their food requirements are no longer met after the age of 7. Death through starvation is thus likely. This reinforces the idea that passive dispersal across the North Pacific basin is definitely not a viable hypothesis for juvenile western Pacific leatherbacks.

## Summary and conclusion

This paper introduces STAMM, a new IBM simulating the dispersal of juvenile sea turtles under the combined effects of simulated oceanic currents and habitat-driven movements. The modeling of habitat-driven movements derives from previous fish movement [17] and habitat models [18]. It remains quite simple as information is still scarce concerning juveniles’ swimming activity, thermal biology and food requirements. Nevertheless, the first active sea turtle dispersal simulation performed with STAMM yields valuable results concerning the impact of simulated habitat-driven movements on the distribution of juvenile leatherbacks in the North Pacific Ocean. While oceanic currents still appear to be the main factor shaping the dispersal area, simulated habitat-driven movements prove to strongly structure the spatial and temporal distribution of the juveniles within this area.

Quite expectedly, simulated habitat-driven movements lead active juveniles to gather in the NPTZ and to undertake seasonal north-south migrations. They swim towards south and warmer waters as winter approaches and come back towards higher latitudes, where food abounds, during spring.

More surprisingly, juveniles in the NPTZ are simulated to swim mostly towards west, that is against the prevailing eastward oceanic currents. The western part of the North Pacific being more productive than its central part, these dominantly westward habitat-driven movements are generated by the zonal gradient of the feeding habitat. The westward swimming tendency only disappears in the easternmost part of the basin (around 135°W) when simulated juveniles finally get attracted towards the rich waters of the California current ecosystem. This westward swimming activity proves to be the main reason why active turtles cross the Pacific more slowly than the passive ones. This slower crossing scenario is in better agreement with the size distribution of leatherbacks incidentally caught by the Hawaii-based pelagic longline fishery [10].

Swimming velocities not only affect the whole Pacific crossing time but also, and more importantly, the residence time and the spatial distribution of simulated juvenile leatherbacks in different parts of the North Pacific basin. The combination of a mean westward swimming speed with an eastward current speed yields a small mean eastward speed on the ground that steadily decreases from the western to the eastern part of the basin. This speed decrease induces higher residence times in the central and eastern part of the basin so that the zone with the largest number of simulated turtle days is located in the NPTZ roughly between the dateline and 135°W. This unfortunately is a zone where the Hawaii longline fishery is particularly active [62] and where the risk of incidental catch shall thus be increased.

Interestingly, Briscoe et al [61] recently observed that juvenile loggerheads also display high residency in the Central North Pacific. They envision different hypotheses possibly explaining this increased residence time, including scenarios relying on navigational markers linked to the Earth magnetic field. Although STAMM is tuned here to simulate the movements of leatherback rather than loggerhead turtles, our results suggest that a simple dispersal model forced only by ocean currents, surface temperatures and NPP fields can produce a similar increase of the residence time in the central and eastern North Pacific.

Besides inducing a generally westward swimming activity, the simulated zonal distribution of the feeding habitats also likely induces important variations in individual fitness. While juveniles likely find sufficient food in the Western North Pacific, they probably know harsh times in the central and eastern part of the basin before finally thriving when reaching the rich California current region. They shall accumulate there large amounts of energy and could then initiate their first reproductive migration. Under that hypothesis, the juvenile western Pacific leatherbacks that cross the Pacific shall reach sexual maturity after 15 years, the mean age at which active turtles reach the California current region. This appears to be a reasonable estimate [53].

Last but not least, the comparison of passive and active dispersal simulations shows that simulated habitat-driven movements strongly reduce the risk of cold-induced mortality. The mortality rate reaches 70% in passive turtles but remains below 20% in active turtles. Simulations also show that cold-induced mortality is more frequent among the simulated juveniles that rapidly circulate into the Kuroshio than among those that first drift into the North Equatorial Counter Current (NECC). The position and strength of the NECC being directly related to El Niño activity [63], this mechanism might induce marked inter-annual variability in juvenile survival. More generally, our results suggest that inter-annual or longer-term variability in ocean currents can induce significant variations in dispersal patterns, leading different proportions of hatchlings, and then juveniles, towards different (more or less favorable) developmental areas. This can, in turn, influence population resilience to regional or global perturbations and possibly allow the emergence of new migration destinations [9]. This shall be further investigated when longer ocean reanalyses, (preferably including multiple El Niño/La Niña events) will become available.

Ultimately, we want to emphasize that the calibration and validation of a sea turtle movement model like STAMM critically depends on the availability of precise information not only concerning juvenile movements but also juvenile thermal biology and energetics. Recent work of Jones and colleagues [22,24,41,44] provide invaluable information on these last two subjects, at least concerning leatherbacks. More work of this type is clearly needed for all sea turtle species. More tracking data are also needed to document movements of young sea turtles. The number of juvenile tracking experiments is steadily growing [4,12,55,61,64,65] but these mostly concern loggerheads. We recommend that such experiments be continued and extended to other sea turtle species, in particular leatherbacks.

## Supporting information

S1 Fig Animated 18-year-long dispersal of passive leatherbacks in the North Pacific Ocean. Simulated individuals are released offshore Jamursba-Medi nesting beach (white dot on the map). Their positions (blue dots) are displayed at 10-day intervals. Dots turn black when cold-induced death occurs.
(AVI)

S2 Fig Animated 18-year-long dispersal of active leatherback and maps of habitat suitability index (*h*) in the North Pacific Ocean. Simulated individuals are released offshore Jamursba-Medi nesting beach (white dot on the map). Their positions (blue dots) are displayed at 10-day intervals. Dots turn black when cold-induced death occurs.
(AVI)
